# “Brain Fog” by COVID-19 or Alzheimer’s Disease? A Case Report

**DOI:** 10.3389/fpsyg.2021.724022

**Published:** 2021-11-04

**Authors:** Jordi A. Matias-Guiu, Cristina Delgado-Alonso, Miguel Yus, Carmen Polidura, Natividad Gómez-Ruiz, María Valles-Salgado, Isabel Ortega-Madueño, María Nieves Cabrera-Martín, Jorge Matias-Guiu

**Affiliations:** ^1^Department of Neurology, Institute of Neuroscience, Hospital Clinico San Carlos, Instituto de Investigación Sanitaria San Carlos (IdISSC), Universidad Complutense, Madrid, Spain; ^2^Department of Radiology, Hospital Clinico San Carlos, Instituto de Investigación Sanitaria San Carlos (IdISSC), Universidad Complutense, Madrid, Spain; ^3^Institute of Laboratory Medicine, Hospital Clinico San Carlos, Instituto de Investigación Sanitaria San Carlos (IdISSC), Universidad Complutense, Madrid, Spain; ^4^Department of Nuclear Medicine, Hospital Clinico San Carlos, Instituto de Investigación Sanitaria San Carlos (IdISSC), Universidad Complutense, Madrid, Spain

**Keywords:** COVID-19, Alzheimer’s disease, neurodegenenerative diseases, neuropsychological assesment, brain imaging

## Abstract

Cognitive symptoms after COVID-19 have been increasingly recognized several months after the acute infection and have been designated as “brain fog.” We report a patient with cognitive symptoms that started immediately after COVID-19, in which cerebrospinal fluid biomarkers were highly suggestive of Alzheimer’s disease. Our case highlights the need to examine patients with cognitive symptoms following COVID-19 comprehensively. A detailed assessment combining clinical, cognitive, and biomarker studies may help disentangle the underlying mechanisms associated with cognitive dysfunction in each case. The investigation of neurodegenerative processes in an early stage, especially in older patients, is probably warranted.

## Introduction

The Severe Acute Respiratory Syndrome Coronavirus 2 (SARS-CoV-2) can affect multiple organs and tissues, including the central nervous system. Post-acute manifestations are included under the umbrella terms of post-COVID-19 syndrome or long-COVID-19 and are relatively common after Coronavirus Disease-2019 (COVID-19; Hellmuth et al., 2021; Nalbandian et al., 2021). Specifically, cognitive issues are among the most frequent neurological symptoms reported by patients after the acute phase (Bliddal et al., 2021; Vanichkachorn et al., 2021). Evidence regarding the prevalence, characteristics, and mechanisms associated with cognitive dysfunction after COVID-19 is still scarce (Daroische et al., 2021). To date, heterogeneous findings have been found in several cognitive domains, especially concerning attention and executive functioning and episodic memory (Almeria et al., 2020; Woo et al., 2020; Zhou et al., 2020). However, studies have used mainly brief cognitive tests or online surveys, which are not designed to characterize the neuropsychological profile associated with COVID-19 (Daroische et al., 2021). Furthermore, most studies do not include a healthy control group, which can hinder obtaining reliable conclusions. The mechanisms underlying cognitive dysfunction after COVID-19 are largely unknown. Hypoxia or vascular damage could explain cognitive deficits, especially in those patients with severe acute infections requiring intensive care and/or respiratory support (Alonso-Lana et al., 2020). Endothelial dysfunction has also been hypothesized, causing microvascular injury (Zhou et al., 2020; Martynov et al., 2021). However, patients with mild infections also report cognitive symptoms (Bliddal et al., 2021). In this regard, some authors have suggested additional mechanisms, including immunological dysregulation, chronic inflammation, or dysfunction of peripheral organs (Moghimi et al., 2021). Other studies have associated cognitive symptoms with anxiety and depression (Almeria et al., 2020).

Additionally, the relationship between COVID-19 and its potential role in future neurodegeneration is currently under debate (Gómez-Pinedo et al., 2020). A few cases of parkinsonism after COVID-19 have been reported so far. Although the possibility of post-infectious parkinsonism cannot be excluded, these cases probably suggest that COVID-19 may unmask an underlying Parkinson’s disease, which was previously in a preclinical stage (Cavallieri et al., 2021; Makhoul and Jankovic, 2021).

We report a patient who developed cognitive symptoms immediately after COVID-19 but showed cerebrospinal fluid biomarkers highly suggestive of Alzheimer’s disease (AD). Our case suggests that COVID-19 could unmask previously preclinical AD, progressing to a prodromal stage. This highlights the need to examine the presence of a neurodegenerative process in older patients reporting cognitive complaints after COVID-19.

## Case Description

**Figure 1:**
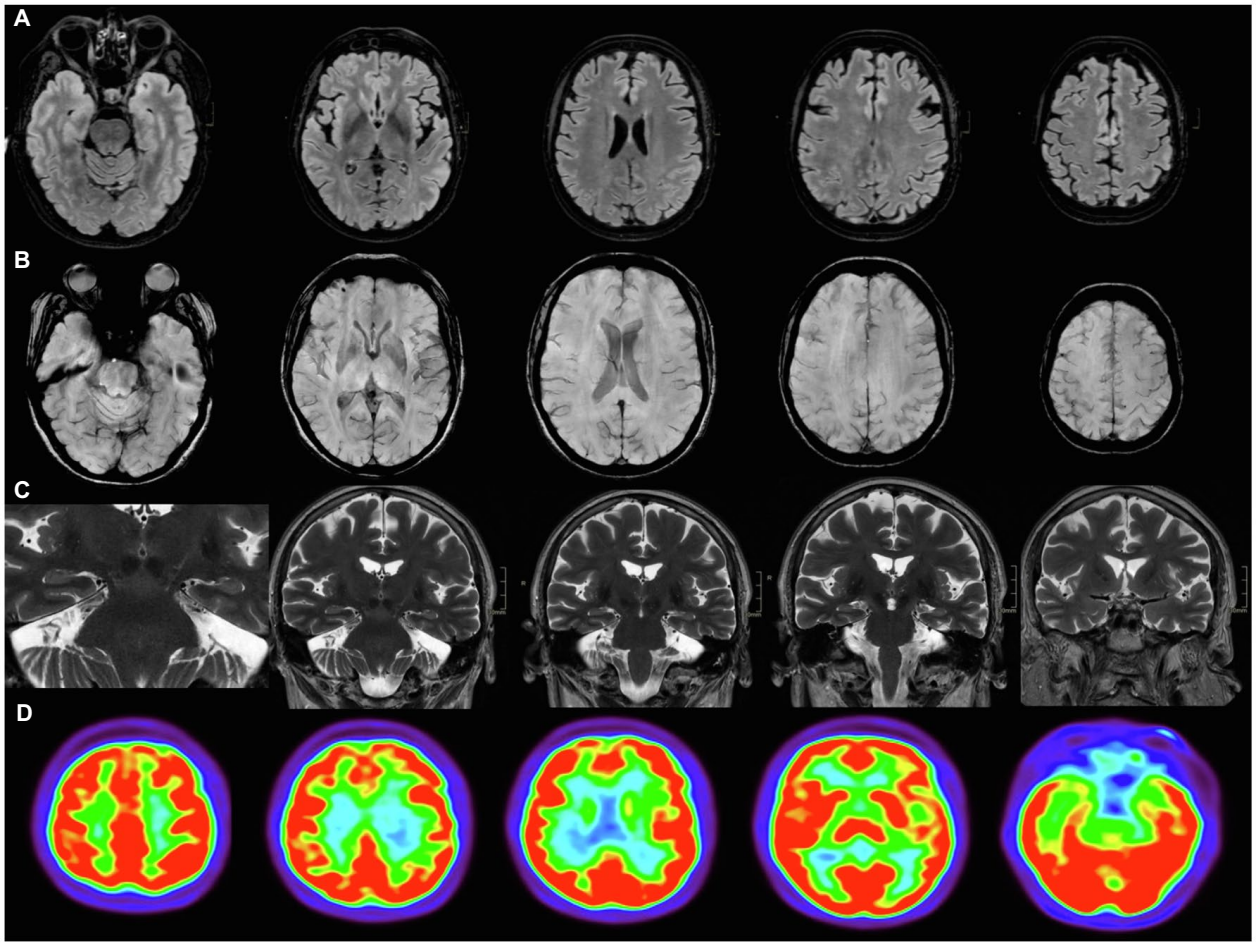
Neuroimaging. Brain MRI **(A–C)** and FDG-PET **(D)**. **(A)** Axial FLAIR, with no white matter hyperintensities; **(B)** axial SWAN, showing no microbleeds; **(C)** coronal FSE T2 PROPELLER, with no hippocampus atrophy or ischemic lesions; and **(D)** FDG-PET showed no regions of hypometabolism visually.

**Figure 2:**
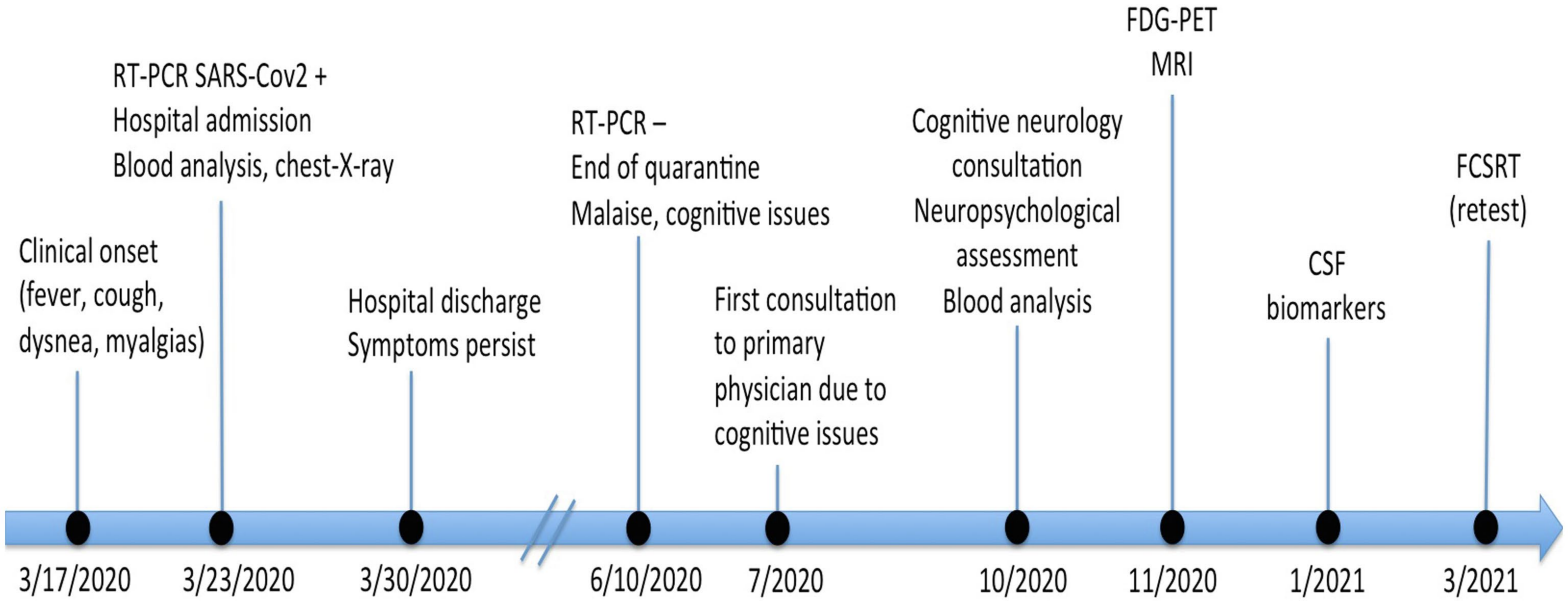
Timeline of events and tests for the case study.

A 67-year-old woman with history of type 2 diabetes mellitus presented with fever, cough, breath difficulties, and myalgias. The patient had no relevant past medical or psychiatric history, except for rheumatic fever in childhood and a benign uterine tumor removed in 2016. There was no family history of dementia or any neurodegenerative disorder. The patient was independent for all advanced, instrumental, and basic activities of daily living. She kept an active lifestyle (for instance, she lived alone, gave lectures, and made social volunteer work). Symptoms started on March 17th 2020, and diagnosis of COVID-19 was confirmed by reverse transcription–polymerase chain reaction (RT-PCR) 6days later. At hospital admission, oxygen saturation was 96%, temperature was 37.1°C, and heart rate 106 beats per minute. Laboratory data showed elevated C-reactive protein, ferritin, and Lactate Dehydrogenase. These are laboratory markers of inflammation often elevated in COVID-19. Furthermore, D-dimer, a fibrin degradation product associated with risk of thrombosis, and frequently increased in the acute phase of the SARS-CoV-2 infection, was also elevated. Chest-X-ray showed bilateral pneumonia (Table 1). She required hospital admission for 7days and was treated with oxygen therapy with a nasal cannula, heparin, and hydroxychloroquine. After she was discharged, she remained at home in quarantine for 90days with persistent generalized malaise for at least 2 months. Two RT-PCR were performed during this period, which remained positive. The first negative RT-PCR was on June 10th. Despite the time elapsed, she complained of persistent cognitive issues and was evaluated in October. These cognitive complaints included memory loss, difficulties in concentration especially during reading, and cognitive fatigue. Both the patient and her family confirmed the temporal relationship between acute COVID-19 symptoms and cognitive symptoms. Clinical Dementia Rating Sum of Boxes at this moment was 1 (Memory 0.5 and Community Affairs 0.5). Retrospective assessment of Clinical Dementia Rating Sum of Boxes previous to COVID-19 was 0. Laboratory data at this time showed no abnormalities, except a mild elevation of C-reactive protein. Hemoglobin A1C was 6.7%. Renal function, iron, B12 vitamin, folate, and thyroid function were within normal limits [Creatinine 0.6mg/dl (normal range 0.5–0.96), Iron 104 micrograms/dl (40–145); B12 vitamin 287pg./ml (180–914); folate 7.43ng/ml (3.1–20.0); and TSH 1.31 micro-international units/ml (0.38–5.33)]. Serological investigations for syphilis and HIV were negative. At this moment, the patient was taken metformine for diabetes mellitus and zolpidem on demand when insomnia. Neuropsychological assessment revealed a verbal episodic memory deficit in two tests (Free and Cued Selective Reminding Test, FCSRT, and Loewenstein-Acevedo Scale for Semantic Interference and Learning, LASSI-L, showing a failure in recovering from proactive semantic interference). The other neuropsychological tests were unremarkable (Table 2). FDG-PET and MRI showed no abnormalities on visual analysis (Figure 1). Semi-quantitative analysis of meta-region of interest (meta-ROI) of FDG-PET was 1.19, indicative of hypometabolism in regions linked to AD (Landau et al., 2011). CSF analysis showed decreased Aβ_1-42_ and elevated tau and phospho-tau. Thus, the patient was diagnosed with AD at a prodromal stage. Six months later, the patients reported some improvement in cognitive symptoms. Memory assessment with a parallel version of FCSRT showed a slight improvement. Timeline of events and tests for the case study are shown in Figure 2. This patient was examined as part of an ongoing study evaluating cognitive impairment after COVID-19, which our local Ethics Committee approved, and the patient gave written informed consent.

**Table 1:** Main laboratory data.

Blood	March 2020	October 2020	Normal range
C-reactive protein	10.70mg/dl	1.01mg/dl	0.1–0.5
Ferritin	448.2ng/ml	70.9ng/ml	30–350
D-dimer	814ng/ml	401ng/ml	0.1–500
Lactate dehydrogenase	616U	362U	250–480
Hemoglobin	14.2g/dl	14.5g/dl	12.0–16.0
White cell count	7,800/mcL	5,500/mcL	4,000-10,500
Platelets	189,000/mcL	223,000/mcL	150,000-450,000
Lymphocytes	800/mcL	1,500/mcL	1,500-3,500
Creatinine	0.73mg/dl	0.62mg/dl	0.51–0.95
Sodium	133mmol/l	142mmol/l	135–45
Potassium	4.0mmol/l	4.1mmol/l	3.4–5.5
Alanine aminotransferase	29U/l	15	5.0–30.0
Aspartate aminotransferase	39U/l	21	5.0–40.0
**CSF**		**January 2021**	
Aβ_1-42_	-	780pg./ml	>900
ratio Aβ_1-42/1–40_	-	0.052	0.068–0.115
Total tau	-	574pg./ml	146–410
Phospho-tau	-	95.4pg./ml	21.5–59.0

**Table 2:** Neuropsychological assessment (67years old, 18years of formal education).

	October 2020		March 2021	
Test	Raw score	Age-, education-adjusted score (percentile)	Raw score	Age-, education-adjusted score (percentile)
**Attention-Executive functioning**				
Trail making test part A	20.15s	41	-	-
Trail making test part B	34.47s	38	-	-
WAF battery (VTS). Time of mean reaction	224ms	54	-	-
N-Back verbal. Number of correct items	14	63	-	-
Symbol Digit Modalities Test	40	50	-	-
Response inhibition (go/no go). Number of errors	4	48	-	-
Cognitrone	3.07	46	-	-
Stroop Color-Word Interference (part A)	100	50	-	-
Stroop color-word interference (part B)	61	35	-	-
Stroop color-word interference (part C, interference)	40	60	-	-
**Constructive praxis and visual perception**				
Rey-Osterrieth complex figure (copy accuracy)	34/35	50	-	-
Rey-Osterrieth complex figure (copy time)	126s	60	-	-
WAF battery (VTS) – Neglect	0	51	-	-
VOSP object decision	19/20	82	-	-
VOSP progressive silhouettes	10	50	-	-
VOSP number location	10/10	98	-	-
Judgment Line Orientation	23/30	50	-	-
**Episodic memory**				
FCSRT free recall trial 1	6/16	50	6	50
FCSRT total free recall	10/48	2	15	5
FCSRT total recall	19/48	<1	28	5
FCSRT delayed free recall	1/16	<1	4	2
FCSRT delayed total recall	8/16	5	8	5
LASSI-L FRA1	10/15	72	-	-
LASSI-L CRA1	7/15	5	-	-
LASSI-L CRA2 (maximum storage)	11/15	15	-	-
LASSI-L FRB1	3/15	10	-	-
LASSI-L CRB1	3/15	5	-	-
LASSI-L CRB2 (recovery from proactive interference)	4/15	<1	-	-
LASSI-L SdFRA	0/15	<1	-	-
LASSI-L SdCRA (retroactive interference)	5/15	10	-	-
LASSI-L delayed recall	9/30	5	-	-
**Language and verbal fluency**				
Boston naming test	50/60	25	-	-
Semantic verbal fluency (animals)	19	65	-	-
Letter fluency (words beginning with “p”	18	25	-	-
**Other questionnaires**				
Brief smell identification test	11/12	Normal	-	-
Modified fatigue impact scale	Total 53/84	Impaired	-	-
Pittsburg sleep quality index	4	Normal	-	-
Beck depression inventory	2	Normal	-	-
State–trait anxiety inventory	State Anxiety: 36 Trait Anxiety: 21	80 (state); 50 (trait); Normal	-	-

Abbreviations: FCSRT, free and cued selective reminding rest; LASSI-L, Loewenstein-Acevedo Scale for semantic interference and learning; VOSP, visual object and space perception battery; WAF battery (VTS): perception and attention functions battery, Vienna Test System.

## Diagnostic Assessments

Cognitive assessments included the following paper and pencil tests: forward and backward digit span, Corsi block-tapping test, Symbol Digit Modalities Test, Boston Naming Test (BNT), verbal fluencies (animals and words beginning with “p” in 1min), Judgment Line Orientation (JLO), Rey-Osterrieth Complex Figure (ROCF; copy), FCSRT, LASSI-L (Crocco et al., 2014), Stroop Color-Word Interference Test, and the Visual Object and Space Perception Battery (VOSP; object decision, progressive silhouettes, and number location). Furthermore, the following tests were administered using the computerized Vienna Test System®: The Trail Making Test (TMT, S1 form), Inhibition Response (INHIB, S13 form; a variant of a go/no go task), N-Back Verbal Test (S1 form), Cognitrone (S11 form; a test to assess attention and concentration through comparison of figures with regard to their congruence), and part of the WAF battery (S1 form) of perception and attention functions (time of mean reaction and neglect). Age- and education-adjusted scores were estimated for each test, and a percentile ≤5 was considered as cognitively impaired (Peña-Casanova et al., 2009; Matias-Guiu et al., 2017). Full cognitive assessment was performed in October 2020. In March 2021, FCSRT was administered again using a parallel version (Grau-Guinea et al., 2021).

In addition, the patient was evaluated with the Brief Smell Identification Test (BSIT), State–Trait Anxiety Inventory (STAI), Beck Depression Inventory-II (BDI-II), Pittsburgh Sleep Quality Index (PSQI), and the Modified Fatigue Impact Scale. The following cutoffs were used according to the literature: BSIT ≤8 was regarded as abnormal olfaction; STAI-S≥40 was considered as clinically significant anxiety; BDI-II ≥19 was used to define moderate- or severe depression; PSQI >5 was regarded as poor sleep quality; and MFIS ≥38 was considered as significant fatigue (Spielberger et al., 1983; Buysse et al., 1989; Beck et al., 1996; Doty et al., 1996; Kos et al., 2005).

MRI images were acquired with a 3T scanner (Signa Architect, GE Healthcare, multichannel (48 channels) head coil. The following sequences were acquired as: (a) 3D CUBE FLAIR T2 axial reconstruction; (b) SWAN 3D axial MinIP reconstruction; (c) 3D MPRAGE T1 axial reconstruction; and (d) coronal FSE T2 PROPELLER.

FDG-PET image was acquired following the European guidelines for FDG-PET neuroimaging in a Siemens Biograph TrueView PET-CT. A dose of 185MBq of FDG-PT was injected after at least 6h of fasting. Glucose level was previously checked to ensure that it was below 150mg/dl. A static PET scan was acquired after the patient remained in sensory rest for 30min. Further details about FDG-PET acquisition are specified elsewhere (Matias-Guiu et al., 2015). Statistical Parametric Mapping version 12 (The Wellcome Trust Centre for Neuroimaging, Institute of Neurology, University College of London) was used to preprocess FDG-PET imaging. Images were normalized to the standard space using a validated template (Della Rosa et al., 2014). Marsbar software was used for ROI analysis, using a meta-ROI proposed for early diagnosis of mild cognitive impairment (MCI) and AD using FDG-PET. Cerebellum was used as reference. This meta-ROI comprised bilateral angular gyrus, bilateral posterior cingulate, and bilateral inferior temporal gyrus and has been associated with high risk of progression from MCI. A cutoff point of 1.249 was used, as recommended (Landau et al., 2011).

A cerebrospinal fluid sample was collected by lumbar puncture (interspace L4-L5) at 9am after overnight fasting. The sample was collected in a 10ml polypropylene tube and processed within the first hour after acquisition. The sample was centrifuged, and volumes of 0.5ml were aliquoted into polypropylene tubes. Storage was kept at −80°C until analysis. Lumipulse G600II automated platform was used for the determination of tau, phospho-tau 181, beta-amyloid 1–42 (Aβ_1-42_), and beta-amyloid 1–40 (Aβ_1-40_). All analyses were performed in our own center. Results of beta-amyloid levels were standardized using certified material of reference (Kuhlmann et al., 2017). Cutoffs determined by the manufacturer Fujirebio were used for data interpretation.

## Discussion

We here report a case of cognitive impairment following COVID-19 infection showing CSF biomarkers indicative of AD. A reduced ability to concentrate and other cognitive difficulties have been recently described in patients after the acute phase of COVID-19 (Wijeratne and Crewther, 2020). These complaints have been designated as “brain fog,” but its characterization and definitive diagnosis have not been accurately described (Kingston et al., 2020). In our case, the finding of an isolated episodic memory deficit, suggestive of hippocampal dysfunction, led to investigate AD biomarkers. According to the current international recommendations, this case meets the diagnostic criteria for prodromal AD and mild cognitive impairment due to AD (Albert et al., 2011; Sperling et al., 2011; Dubois et al., 2014). In this regard, some findings, such as an episodic memory deficit with preserved attention, a low benefit through category cues during controlled learning tests for episodic memory assessment, or failure to recover from proactive semantic interference (closely associated with amyloid deposition and AD; Loewenstein et al., 2018), should be considered as patterns highly suggestive of Alzheimer’s pathology, as we observed in this patient. The low benefit of semantic cues during episodic memory tests using a controlled memory encoding procedure with semantic cues along several trials is regarded as suggestive of hippocampal dysfunction, as seen in AD. Accordingly, this cognitive marker is helpful to differentiate AD from other causes of memory dysfunction related to fronto-striatal dysfunction, such as depression, vascular cognitive impairment, or other neurodegenerative conditions (Teichmann et al., 2017). Furthermore, the LASSI-L, a novel and challenging cognitive test, has shown promising results in detecting the earliest changes of AD in prodromal and preclinical stages. This test evaluates the ability to learn a first list of 15 words on three semantic categories during two trials. Then, a second list of 15 different words from the same semantic categories is also administered during two trials. This strategy allows the assessment of proactive semantic interference and, uniquely, the recovery from proactive semantic interference, which has been identified as a sensitive AD marker in several studies (Loewenstein et al., 2016; Matias-Guiu et al., 2018; Abulafia et al., 2019). Subsequently, a free and cued recall of the first list is performed to evaluate retroactive semantic interference. Finally, delayed recall is evaluated at 30min. The neuropsychological findings in our case were highly suggestive of AD in an early stage. Although the “brain fog” associated with COVID-19 seems to emphasize the attention deficits, the multiple pathophysiological processes associated with COVID-19 potentially causing cognitive impairment (hypoxia, neuroinflammation, systemic involvement, etc.) probably suggest that several cognitive profiles may be expected in these patients; this would also explain the heterogeneous findings reported so far (Hampshire et al., 2021; Raman et al., 2021). Thus, well-designed studies examining cognitive functions in patients after COVID-19 comprehensively are necessary to describe the neuropsychological characteristics and the best discriminators from other causes of cognitive impairment.

This case illustrates the need for a thorough assessment of patients reporting cognitive complaints after COVID-19. It is true that some patients exhibit cognitive issues following the acute infection, although the specific mechanisms that explain these symptoms are still unclear. A possible explanation could be the brain damage associated with hypoxia. In this regard, our patient required oxygen therapy through a nasal cannula for several days. Patients showing severe brain hypoxia, such as cardiac arrest, usually show memory deficits and psychomotor slowing, or global deficits in all cognitive domains (Alexander et al., 2011). The rationale for this cognitive profile is the high sensitivity of hippocampus and basal ganglia to hypoxia (Haglund et al., 2019). Although it has not been elucidated, this cognitive picture could be expected in patients with COVID-19 with severe respiratory involvement. In our case, psychomotor tests (e.g., TMT part A and Symbol Digit Modalities Test) were within normal limits, and there were no hypoxic changes in neuroimaging. However, microstructural changes associated with mild degrees of hypoxia contributing to the cognitive disorder cannot be excluded.

Considering the clinical and cognitive characteristics and the findings from neuroimaging and CSF biomarkers, we believe that COVID-19 could unmask cognitive symptoms in a patient in a previous preclinical stage of AD, progressing to amnestic mild cognitive impairment due to AD. However, AD diagnosis should be confirmed by clinical follow-up and neuropathological examination. Greater susceptibility to COVID-19 issues in patients with AD has been suggested due to a differential expression of ACE2 in the brain of these patients, with a lower response to oxidative stress (Ding et al., 2021). Furthermore, Apolipoprotein Ee4 genotype is associated with an increased risk of AD and COVID-19 severity (Matias-Guiu et al., 2020). In addition, an indirect effect of COVID-19 on amyloid-β, tau, and TDP-43 pathology has also been suggested (Minners et al., 2020). An activation of renin-angiotensin system in COVID-19 could also induce changes in brain beta-amyloid and tau levels (Minners et al., 2020). Recent investigations have reported similarities between the transcriptomic analysis in the frontal cortex of patients who died from COVID-19 and neurodegenerative disorders, with abnormal activation of astrocytes and microglia (Yang et al., 2021). Besides, SARS-CoV-2 and other coronaviruses can remain latent in neurons, and it may hypothetically induce protein misfolding and aggregation (Fotuhi et al., 2020; Nath, 2020). Thus, further knowledge on the role of COVID-19 in the pathophysiology of neurodegenerative disorders is urgently needed.

The main limitation of this case report is the absence of a cognitive examination before COVID-19. However, retrospective interviews with the patient and relatives found no cognitive symptoms. Furthermore, the initial cognitive worsening during the acute infection and the partial recovery observed during the subsequent months support the hypothesis of the unmasking effect of COVID-19 over a latent preclinical neurodegenerative disorder. In this regard, memory scores of the FCSRT remained impaired (e.g., adjusted scores below percentiles ≤5), confirming the persistence of cognitive dysfunction, which is according with the diagnosis of prodromal AD.

In conclusion, patients with cognitive complaints following COVID-19 should be comprehensively examined. An accurate description of COVID-19-related persistent cognitive symptoms, and the best cognitive hallmarks to differentiate it from other degenerative entities, is necessary for an adequate differential diagnosis. A detailed assessment combining clinical, cognitive, and biomarker studies may help disentangle the underlying mechanisms associated with cognitive dysfunction in each case. The investigation of neurodegenerative processes in an early stage, especially in older patients reporting cognitive complaints after COVID-19, is probably warranted.

## Data Availability Statement

The raw data supporting the conclusions of this article will be made available by the authors, without undue reservation.

## Ethics Statement

The studies involving human participants were reviewed and approved by Comité de Ética e Investigación Clínica Hospital Clinico San Carlos. The patients/participants provided their written informed consent to participate in this study. Written informed consent was obtained from the individual(s) for the publication of any potentially identifiable images or data included in this article.

## Author Contributions

JAM-G, JM-G, and MY: conceptualization. CD-A, MV-S, NG-R, CP, MC-M, and IS-M: data curation. JAM-G: funding acquisition and writing original draft. JAM-G and MY: methodology. JM-G and MC-M: supervision. JAM-G, CD-A, MY, CP, NG-R, MV-S, IS-M, MC-M, and JM-G: writing review and editing. All authors contributed to the article and approved the submitted version.

## Funding

JAM-G is supported by the Instituto de Salud Carlos III through the project INT20/00079 (co-funded by European Regional Development Fund “A way to make Europe”). MV-S is supported by the Instituto de Salud Carlos III through a predoctoral contract (FI20/000145; co-funded by European Regional Development Fund “A way to make Europe”).

## Conflict of Interest

The authors declare that the research was conducted in the absence of any commercial or financial relationships that could be construed as a potential conflict of interest.

## Publisher’s Note

All claims expressed in this article are solely those of the authors and do not necessarily represent those of their affiliated organizations, or those of the publisher, the editors and the reviewers. Any product that may be evaluated in this article, or claim that may be made by its manufacturer, is not guaranteed or endorsed by the publisher.
